# Psychological complications in patients with acromegaly: relationships with sex, arthropathy, and quality of life

**DOI:** 10.1007/s12020-022-03106-8

**Published:** 2022-07-02

**Authors:** Biagio Cangiano, Emanuele Giusti, Caterina Premoli, Davide Soranna, Giovanni Vitale, Silvia Grottoli, Valeria Cambria, Giovanna Mantovani, Roberta Mungari, Pietro Maffei, Francesca Dassie, Antonella Giampietro, Sabrina Chiloiro, Maria Laura Tanda, Silvia Ippolito, Salvatore Cannavò, Marta Ragonese, Antonella Zambon, Luca Persani, Letizia Maria Fatti, Massimo Scacchi, on behalf of the “PRO-ACRO” study group on Motor Disability in Acromegaly, of the Italian Society of Endocrinology (SIE), Francesco Cavagnini, Diego Ferone, Sabrina Corbetta, Luigi Bartalena, Paolo Beck Peccoz, Maura Arosio, Andrea Lania, Annamaria Colao, Rosario Pivonello, Ettore Degli Uberti, Ezio Ghigo, Andrea Giustina, Enio Martino, Alfredo Pontecorvi, Nicola Sicolo, Francesco Trimarchi

**Affiliations:** 1 Division of Endocrine and Metabolic Diseases, Istituto Auxologico Italiano IRCCS, Milan, Italy; 2 Department of Medical Biotechnology and Translational Medicine, University of Milan, Milan, Italy; 3 Psychology Research Laboratory, IRCCS Istituto Auxologico Italiano, P.le Brescia 20, Milan, Italy; 4 Department of Psychology, Catholic University of the Sacred Heart, Milan, Italy; 5 Istituto Auxologico Italiano, IRCCS, Milan, Italy; 6 Department of Medical Sciences, University of Turin, Turin, Italy; 7 Endocrine Unit, Fondazione IRCCS Ca’ Granda Ospedale Maggiore Policlinico, Milan, Italy; 8 Department of Clinical Sciences and Community Health, University of Milan, Milan, Italy; 9 Department of Medicine-DIMED, University of Padua, Padua, Italy; 10 Pituitary Unit, Section of Endocrinology, Department of Internal Medicine, Catholic University, A. Gemelli University Hospital, Rome, Italy; 11 Department of Clinical and Experimental Medicine, University of Insubria, Varese, Italy; 12 Department of Human Pathology, University of Messina, Messina, Italy; 13 Endocrine Unit, University Hospital of Messina, Messina, Italy; 14 Division of Biostatistics, Epidemiology and Public Health, Department of Statistics and Quantitative Methods, University of Milano-Bicocca, Milan, Italy; 15 Istituto Auxologico Italiano, IRCCS, Division of General Medicine, Ospedale S. Giuseppe, Oggebbio-Piancavallo, Verbania, Italy; 16 Neuroendocrinology Research Laboratory, Istituto Auxologico Italiano IRCCS, Cusano Milanino, Italy; 17 Endocrinology Unit, IRCCS Ospedale Policlinico San Martino, 16132 Genoa, Italy; 18 Endocrinology Unit, Department of Internal Medicine and Medical Specialties (DIMI), University of Genoa, 16132 Genoa, Italy; 19 Endocrinology and Diabetology Service, IRCCS Istituto Ortopedico Galeazzi, Milan, Italy; 20 Department of Medicine and Surgery, University of Insubria, Varese, Italy; 21 Fondazione Istituto di Ricovero e Cura a Carattere Scientifico Cà Granda Ospedale Maggiore Policlinico, Via Pietro Custodi 16, 20136 Milano, Italy; 22 Department of Clinical Sciences and Community Health, University of Milan, 20122 Milan, Italy; 23 Department of Biomedical Sciences, Humanitas University, Pieve Emanuele, 20090 Milan, Italy; 24 Department of Clinical Medicine and Surgery, Endocrinology Unit, Federico II University, Naples, Italy; 25 UNESCO Chair on Health Education and Sustainable Development, Federico II University, Naples, Italy; 26 Department of Medical Sciences, Section of Endocrinology and Internal Medicine, University of Ferrara, Ferrara, Italy; 27 Department of Medical Sciences, University of Torino, 10126 Torino, Italy; 28 Institute of Endocrine and Metabolic Sciences, Università Vita-Salute San Raffaele, IRCCS Ospedale San Raffaele, Milan, Italy; 29 Unit of Endocrinology, Department of Clinical and Experimental Medicine, University of Pisa, Pisa, Italy; 30 Division of Endocrinology, Fondazione Policlinico Universitario “Agostino Gemelli” – IRCCS, Rome, Italy; 31 Department of Medicine (DIMED), Clinica Medica 3^, University of Padua, Via Giustiniani, 2, 35124 Padua, Italy; 32 Accademia Peloritana dei Pericolanti, University of Messina, Messina, Italy; 33 Department of Clinical and Experimental Medicine, University of Messina, Messina, Italy

**Keywords:** Acromegaly, Depression, Articular function, QoL, Anxiety, Radiotherapy

## Abstract

**Purpose:**

Current treatment of acromegaly restores a normal life expectancy in most cases. So, the study of persistent complications affecting patients’ quality of life (QoL) is of paramount importance, especially motor disability and depression. In a large cohort of acromegalic patients we aimed at establishing the prevalence of depression, to look for clinical and sociodemographic factors associated with it, and to investigate the respective roles (and interactions) of depression and arthropathy in influencing QoL.

**Methods:**

One hundred and seventy-one acromegalic patients (95 women and 76 men, aged 20–85 years) among those recruited in a cross-sectional Italian multicentric study were investigated. Each patient filled in three validated questionnaires: AcroQoL, WOMAC (measuring articular pain, stiffness and functionality), and AIMS (evaluating articular symptoms and depression).

**Results:**

A very high (up to 28%) depression rate was detected in acromegalic subjects. Two patients showing pathological AIMS depression scores, committed suicide during the three years observational period. In our population poor psychological status was significantly associated with female sex. Furthermore, a significant strong correlation was found between AIMS depression score and WOMAC score. Both depression and arthropathy-related motor disability turned out to independently contribute with similar strength to the impairment of QoL.

**Conclusions:**

We report a high prevalence of depression in acromegaly, which is associated with female sex and arthropathy. Both depression and arthropathy strongly and independently contribute to the impaired QoL of patients. Our study shows that assessment and monitoring of psychological status is mandatory in acromegaly, also suggesting an inexpensive tool for this assessment.

## Introduction

The excess of growth hormone (GH) characterizing acromegaly causes a systemic disease affecting a great number of organs and tissues [1, 2]. If the treatment is ineffective, the patient can experience cardiovascular, cerebrovascular, neoplastic, respiratory or metabolic complications, whose consequences range from reduction of life expectancy to important life-threating conditions, according to the severity of the disease [2, 3]. Other chronic complications such as acromegalic arthropathy, even if not influencing patients’ survival, heavily affect subjects’ quality of life (QoL) [4]. These complications are often permanent despite the remission of the acromegalic disease (and physical rehabilitation). QoL is also affected by diagnostic delays and patient-related factors, such as age and body mass index [5]. Acromegalic patients face various psychological issues, which can potentially undermine their QoL. They have a prevalence of psychiatric comorbidity between 40 and 50% [6, 7], as well as an increased prevalence of affective disorders (in particular depression) compared to both healthy controls and controls with other chronic disorders [6, 8]. Furthermore, they might have a disrupted body image [9–11] and are more likely to present anxiety-related personality traits, such as harm avoidance and neuroticism [12]. Depression is known as a major determinant of QoL in acromegalic patients [13]; however, it has been often evaluated in combination with other psychological and psychiatric disorders [6–8], so that few data are available regarding major depression singularly.

The management of acromegaly greatly improved during the last decades, allowing the remission of the disease in the majority of subjects and restoring life expectancy to normal levels [2, 3]. Thus, the study of persistent complications affecting patients’ QoL has become of paramount importance. Yet, to date, few studies investigated the effects of the different therapeutic strategies used in acromegaly on the development of major depression, and no study focused on the impact of chronic pain and disability deriving from acromegalic arthropathy on psychological complications. Moreover, it is not known which one between depression and arthropathy plays a major role in the impairment of patients’ well-being, and if they are mutually influenced.

The aims of the present study are therefore: (1) to quantify the prevalence and the degree of depression in acromegalic patients using a simple tool, (2) to look for clinical and sociodemographic factors associated with psychological complications in the acromegalic disease, and (3) to investigate the role of depression, adjusted for articular function, on QoL.

## Methods

### Patients

In this retrospective analysis we included one hundred and seventy-one acromegalic subjects (76 men and 95 women, age 20–85 years) without inclusion restriction based on age, sex, treatment or acromegalic disease status, who filled in psychologic questionnaires (see subsection “Questionnaires”) during a cross-sectional, Italian, multicentric investigation of acromegalic arthropathy and QoL [4]. Diagnosis was based on high IGF1 levels, and/or basal/random GH levels >1.0 ng/mL and/or unsuppressed GH after oral glucose tolerance test (see subsection “Hormonal evaluation”). At the inclusion, patients were divided according to normal IGF1 and/or GH levels, in those showing biochemically controlled disease and those with active disease. Given the observational nature of the study, the therapeutic approaches of the different physicians were chosen according to their expertise, with no influence from this study. Yet, surgery was the first-line therapy in the 52.6 % of patients, SSA in the 36.2%, while other therapies and RT were only adopted when the first-line treatments were not effective or viable. In 11.2% of patients, it is not known whether it was initially performed surgery or medical therapy.

Table 1 reports the treatment received by patients with active and controlled disease.

**Table 1:** Treatment received by patients with active and controlled disease

Therapy	Number
Patient with controlled disease (112)	
Previous surgical intervention (4 multiple)	72
Previous RT	18 (5 non-stereotactic RT)
SSA	76
DA	13
Pegvisomant	23
*Among these:*	
SSA + Pegvisomant	6
SSA + DA	11
Pegvisomant +DA	3
SSA + Pegvisomant+ DA	0
Patients with active disease (59, 3 newly diagnosed)	
Previous surgical intervention	18 (5 multiple)
Previous RT	8 (3 non-stereotactic RT)
SSA	30
DA	7
Pegvisomant	8
*Among these:*	
SSA + Pegvisomant	4
SSA + DA	5
Pegvisomant +DA	2
SSA + Pegvisomant+ DA	1

*SSA* somatostatin analogues, *DA* dopamine agonists, *RT* radiation therapy

For each patient we recorded the following potential determinants of psychological complications: age, sex, BMI, time lapsed from diagnosis calculated as the time interval from diagnosis to study enrollment, standardized IGF1 at observation, GH at observation, pituitary defects, diabetes mellitus, medical treatments, fractionated radiotherapy, radiosurgery and surgery. Moreover, as potential confounders we recorded the concomitant use of anti-depressant drugs (SSRI), benzodiazepines, as well as the conditions of hypogonadism and menopause, determining low sex steroids.

Since data were collected three years ago, we also had the opportunity to investigate the three years mortality rate from the start of observation, and the percentage due to suicides. The study was approved by the Institutional Review Board (Istituto Auxologico Italiano, 02C201). All patients gave the informed consents for the use of the anonymized clinical and biochemical data for research and publication purposes.

### Questionnaires

Each patient was administered three validated questionnaires: the AcroQoL (a quality of life continuous scale) [14–16]; Western Ontario and McMaster universities Osteoarthritis Index (WOMAC) [17, 18], composed of three scales evaluating articular pain, stiffness and functionality; and the Arthritis Impact Measurement Scale (AIMS) [19, 20], evaluating both articular symptoms and subject’s depression and anxiety as a continuous scale. Cutoffs for AIMS depression scale to identify possible depression (>3/10) and probable depression (>4/10) were used according to the data published by Hawley et al [21].

### Hormonal evaluation

To avoid the bias associated with different assays used in various laboratories, IGF1 was considered both as absolute value and as estimated standard deviations (eSDs). Assuming that IGF1 values are distributed in a normal pattern, the data of each measurement was standardized using the assay specific reference ranges for age derived from the general population. In particular, we used one of the methods reported by Christy Chuang-Stein in 1992 [22], that considers both the upper (97.5th) and the lower (2.5th) percentiles of the general population, as reported by each manufacturer.

The standardized values were obtained as follows:

$${{{\mathrm{IGF}}}}1_{{{{\mathrm{eSD}}}}} = \left( {{{{\mathrm{IGF}}}}1 - {{{\mathrm{mean}}}}} \right){{{\mathrm{/eSD}}}}$$

where eSD = (mean-lower reference value)/2, and mean = (higher reference value + lower reference value)/2. GH values were not standardized.

### Statistical analysis

The percentage of patients with probable depression (AIMS depression > 4) was calculated, and Chi-Square analyses were performed to compare this prevalence with the percentage of subjects having AIMS scores compatible with probable depression among: patients with rheumatoid arthritis (RA), patients with primary osteoarthritis (OA) of the hip and or knee, patients with low back pain, patients with primary OA of the hand, and other younger patients of the Wichita Arthritis Center among year 1981 and 1991; all these comparative data were derived from literature [21]. We could not perform the same analyses for AIMS anxiety scores, since similar data were not available in the literature.

Determinants of continuous normal outcome were explored by linear regression model. Normality of model residuals was evaluated by graphical and formal test (Shapiro-Wilks). In case of non-normal data, we applied a log-gamma regression model adapted for skewed distribution. Dichotomous frequent outcome was analyzed by log-poisson with robust variance regression model.

For each outcome we considered 3 models: univariate, partial and full multivariate. In the univariate model, we considered only one covariate as determinant of outcome; in the partial model we included all potential determinants; in the full model we added the potential confounders. The effect of covariates on the outcome was reported as regression coefficient (β), its standard error (SE) and *p*-value testing the null hypothesis β = 0.

To evaluate the determinants of psychological complications, we applied the following models: (i) linear regression model for “AIMS Anxiety”, (ii) log-gamma regression model for “AIMS depression”.

The impact of psychological and articular components on quality of life was estimated by linear regression model. To identify potential confounders of these relationships we performed univariate analysis and we included in multivariate model all covariates with *p*-value < 0.20. Standardized regression coefficients were reported to allow a direct comparison between covariates impact.

## Results

**Fig. 1:**
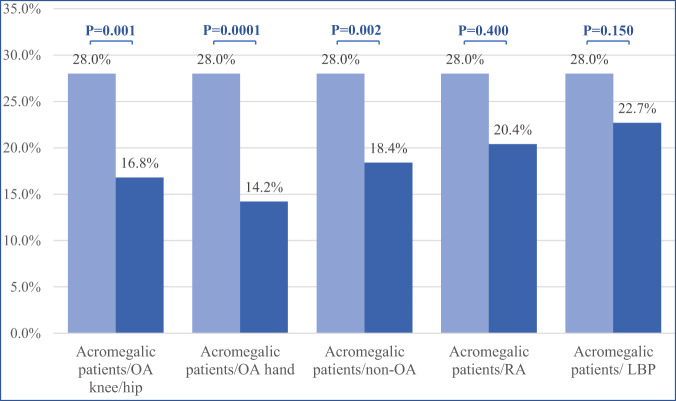
Prevalence of depression in the cohort of acromegalic patients compared to rheumatologic patients without major disability. *RA* Rheumatoid Arthritis*, OA* Osteoarthritis*, LBP* Low back pain*.* Cutoffs for AIMS depression scale to identify probable depression (>4/10) were used (see Questionnaires section). The control groups were taken from the rheumatologic patients without major disability from Wichita Arthritis Center among year 1981 and 1991 (see Statistical Analysis section)

According to the AIMS depression scale 28% of patients with acromegaly displayed probable depression. The proportion was significantly higher than those reported in subjects with primary OA of the hand (14.2%; *p*-value = 0.0001) and in patients without OA (18.4%; *p*-value 0.002). The probable depression in our study population was also significantly higher than that observed in patients with OA of the hip or knee (*p*-value = 0.001), but did not attain statistical significance in the comparison with subjects with RA (p-value = 0.4) and those with low back pain (*p*-value = 0.15) (Fig. 1).

The three years mortality in our sample was 4.67% (Supplementary Table 2). When evaluating the prevalence of suicides in a three-year observation, two patients out of 171 committed suicides, thus not achieving any significant result (Fisher’s exact test), even if both these subjects had pathological AIMS depression scores (4.6/10 and 6.3/10).

Univariate regression models showed that female sex was statistically associated with worse anxiety (*p*-value < 0.001) and depression (*p*-value < 0.001) levels. A statistically significant positive association was reported for radiosurgery and anxiety level (*p*-value = 0.026). No other covariates showed significant associations with outcomes of interest. The associations between sex and both anxiety and depression highlighted by univariate models were confirmed in partial and full (to adjust the data for these possible confounders) multivariate models, while radiosurgery was no longer a determinant of anxiety level after the adjustments (Table 2). Finally, the use of disease activity (active disease vs controlled disease) instead of IGF1eSD, and of the time of active disease (from diagnosis to controlled disease, 5.82 ± 8.1 years) instead of time lapsed from diagnosis (11.53 ± 10.5 years), did not change the results.

**Table 2:** Univariate and multivariate regression models to estimate the effect of exposure covariates on anxiety and depression. Estimates are reported as coefficient regression (β), standard error (SE) and relative *p*-value

			Anxiety AIMS *(*N* = 164)						Depression AIMS **(*N* = 164)			
	Univariate models		Partial multivariate model		Full multivariate model		Univariate models		Partial multivariate model		Full multivariate model	
	β (SE)	*P*-value	β (SE)	*P*-value	β (SE)	*P*-value	β (SE)	*P*-value	β (SE)	*P*-value	β (SE)	*P*-value
Exposure												
Age	−0.010 (0.013)	0.475	−0.016 (0.015)	0.263	−0.02 (0.016)	0.190	0.003 (0.006)	0.544	0.000 (0.006)	0.942	0 (0.007)	0.994
Gender (F vs M)	−1.625 (0.339)	**<0.001**	−1.748 (0.364)	**<0.001**	−1.528 (0.476)	**0.002**	−0.550 (0.158)	**0.001**	−0.580 (0.164)	**0.001**	−0.671 (0.218)	**0.002**
BMI	0.010 (0.034)	0.782	0.025 (0.035)	0.476	0.027 (0.035)	0.440	0.011 (0.015)	0.486	0.01 (0.017)	0.572	0.01 (0.017)	0.544
Time lapsed from diagnosis	0.028 (0.017)	0.101	0.026 (0.021)	0.223	0.022 (0.021)	0.313	0.013 (0.007)	0.089	0.008 (0.01)	0.409	0.007 (0.01)	0.495
Standardized IGF1 at observation	−0.017 (0.045)	0.709	0.011 (0.046)	0.818	0.008 (0.046)	0.867	−0.018 (0.02)	0.376	−0.015 (0.021)	0.478	−0.015 (0.02)	0.462
Pituitary defects	−0.305 (0.378)	0.422	−0.398 (0.384)	0.301	−0.43 (0.385)	0.266	0.005 (0.17)	0.976	−0.095 (0.177)	0.591	−0.134 (0.176)	0.448
Diabetes	−0.292 (0.477)	0.542	−0.399 (0.491)	0.418	−0.441 (0.495)	0.375	0.155 (0.214)	0.469	0.052 (0.233)	0.824	0.01 (0.233)	0.965
Fractionated radiotherapy (Yes vs No)	−0.319 (0.575)	0.580	−0.16 (0.575)	0.781	−0.103 (0.578)	0.859	−0.002 (0.258)	0.994	−0.008 (0.268)	0.976	0.044 (0.268)	0.871
Radio surgery (Yes vs No)	1.846 (0.822)	**0.026**	0.862 (0.909)	0.344	1.027 (0.914)	0.263	0.660 (0.371)	0.077	0.356 (0.415)	0.392	0.444 (0.416)	0.287
Surgery (Yes vs No)	0.036 (0.364)	0.922	0.408 (0.388)	0.294	0.426 (0.391)	0.277	−0.003 (0.163)	0.984	0.043 (0.174)	0.808	0.017 (0.173)	0.920
*Confounders*												
SSRI (Yes vs No)	-	-	-	-	1.035 (0.768)	0.180	-	-	-	-	0.506 (0.36)	0.162
Benzodiazepine (Yes vs No)	-	-	-	-	0.316 (0.748)	0.673	-	-	-	-	0.13 (0.338)	0.701
Low sex hormones (Yes vs No)	-	-	-	-	0.382 (0.474)	0.423	-	-	-	-	−0.082 (0.227)	0.717

*P-*values < 0.05 are reported in bold, *Linear regression model, **Log-gamma regression model

When considering the value of GH at the observation, our cohort was reduced to 131 patients due to missing data. Performing the evaluation both in the complete cohort without considering the GH values, and in the reduced group including this parameter, the results did not change. They remained statistically significant even after considering the confounders (use of SSRI, benzodiazepines, and low sexual hormones).

AIMS-derived depression also showed a strong correlation with WOMAC questionnaire (β 0.45 *p*-value < 0.0001).

The analyses aimed at understanding the impact of depression and arthropathy on quality of life in acromegaly showed that both these parameters are strictly linked to patients’ well-being with similar strength, and high significance (β 0.443 and 0.449 respectively, *p* < 0.001) in both univariate and multivariate models (Table 3). The other parameters associated with AcroQoL had all lower β values (Table 3) and only BMI maintained significance, besides depression and arthropathy, in the multivariate model.

**Table 3:** Univariate and multivariate regression models to estimate the effect of Womac and depression levels on quality of life. Estimates are reported as standardized coefficient regression (β), standard error (SE) and relative *p*-value

			Acro QoL (*N* = 164)	
	Univariate models		Multivariate model	
	β (SE)	*P*-value	β (SE)	*P*-value
Age	−0.067 (0.079)	0.397	-	-
Gender (F vs M)	0.404 (0.072)	**<0.001**	0.139 (0.072)	0.054
BMI	−0.246 (0.076)	**0.002**	−0.142 (0.056)	**0.012**
Time lapsed from diagnosis	−0.168 (0.077)	**0.032**	0.103 (0.06)	0.092
Standardized IGF at observation	0.09 (0.078)	0.25	-	-
Pituitary defects	−0.077 (0.078)	0.325	-	-
Diabetes	−0.065 (0.078)	0.411	-	-
Fractionated radiotherapy (Yes vs No)	−0.042 (0.078)	0.591	-	-
Radiosurgery (Yes vs No)	−0.187 (0.077)	**0.016**	−0.025 (0.058)	0.662
Surgery (Yes vs No)	−0.021 (0.079)	0.793	-	-
Womac total	−0.655 (0.059)	**<0.001**	− 0.408 (0.067)	**<0.001**
Depression AIMS	−0.647 (0.06)	**<0.001**	−0.42 (0.06)	**<0.001**
SSRI (Yes vs No)	0.013 (0.078)	0.871	-	-
Benzodiazepine (Yes vs No)	−0.121 (0.078)	0.121	0.017 (0.052)	0.746
Low sex hormones (Yes vs No)	−0.275 (0.076)	**<0.001**	0.028 (0.067)	0.677

Significant results are shown in bold

The mean and median scores obtained by our cohort to the questionnaires are reported in Supplementary Table 1.

## Discussion

In our series of patients with acromegaly the prevalence of AIMS questionnaires-derived depression was particularly high (28%). The comparison with data from literature [21] reporting rheumatologic patients without major motor disability, showed a significantly worse psychologic condition in acromegalic subjects. When comparing our patients with those carrying more disabling rheumatological diseases, the only significant difference was observed in comparison with subjects with OA of the hip/knee. This finding, if on the one hand confirms an overall increased depression in our cohort, on the other hand cannot fully clarify whether this is mainly due to the disabling arthropathy or it is independent of it. The first hypothesis is suggested by the strict association between psychological status and the severity of arthropathy emerged in our series. In any case, the report of two suicides among subjects with acromegaly scoring more than 4/10 in AIMS depression questionnaire, even if not significant per-se, requires further investigations in other large cohorts of subjects for longer periods. It is worth noting the young age of suicidal deaths compared to the other causes of death (Supplementary Table 2). On a whole these data suggest this questionnaire as an inexpensive tool to screen severe depression in acromegaly.

Apart from the already quoted association of psychological status with arthropathy, the only statistically significant correlation found when evaluating the factors associated with depression and anxiety was female sex. Differently from what we found studying acromegalic arthropathy [4], BMI, standardized IGF1 levels, and diabetes mellitus showed no correlation with anxiety and depression, as measured with our tools, and do not seem to be major determinants of this condition. On the contrary, the lack of association of psychological status with pituitary hormonal defects is possibly due to replacement therapies started in all patients receiving a diagnosis of hypopituitarism. All the results were confirmed adjusting for possible confounders. Our findings showed to be independent from concomitant therapies with SSRI and benzodiazepines. However, given the cross-sectional design of the study, we cannot exclude a role of these treatments in modulating anxiety and depression in patients affected with acromegaly, and prospective studies as well as clinical trials, are needed to provide this information. The adjustment made according to low sex hormone levels (which includes both pathologic hypogonadal status and menopause) did not change our results and the significant difference among sexes. This suggests that the association of female sex with worse depression and anxiety scores is not due to post-menopausal changes. In fact, the interactions between the somatotropic and the gonadal axes are complex, with both a central stimulatory effect and a peripheral liver resistance to GH mediated by estradiol, leading to different hormonal profiles in male and female subjects [23]; also, a different social and psychological impact of the acromegaly-related facial changes could justify this sex gap in anxiety and depression scores.

According to the existing literature, previous treatments represent another factor associated with depression. RT [6, 8] is known to correlate with worse depression scores, probably due to a more severe disease status in patients undergoing this treatment, rather than indicating a direct relationship. Sievers and colleagues also reported that ongoing medical therapy is associated with major depression [6, 8]; however, since there was no adjustment for disease duration, an increased time lapsed from diagnosis in patients still assuming medical therapies could explain this correlation. In our study, we found RT to be significantly correlated only with worse anxiety scores and this association was lost in the multivariate model. This could be explained with a lesser damage to the remaining pituitary using stereotactic technologies and with the minimal number of patients who underwent fractionated RT in our cohort (*n* = 8).

**Fig. 2:**
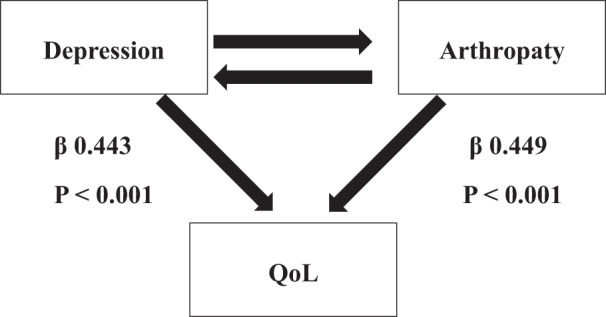
Correlation between depression and arthropathy on quality of life in acromegalic patients

The evaluation of the impact of depression and arthropathy on QoL in patients with acromegaly showed a strong correlation of both these variables with a worse well-being. The presence of an association between depression and QoL confirms findings of other smaller studies [24]. The strong association of psychologic and osteoarticular complications can be due to a bidirectional cause-effect: it is known that chronic pain has a negative effect on mood disorders and, the other way around, depressed patients are more inclined to score higher to pain scales, having a decreased tolerance to pain. More, our data show that, analyzing these variables together, their association with impaired QoL remains significant, probably because each condition has an independent disrupting potential on patients’ well-being. (Fig. 2). It has to be mentioned that also fractures and osteoporotic complications, highly prevalent in active acromegaly [25], could affect WOMAC scores and, since a standardized evaluation of vertebral morphometry in our cohort was not carried out, we can not rule out fractures as determinants of worse “articular” scores.

The implications of our findings are twofold. Firstly, it is important to assess and monitor the psychological status of patients affected with acromegaly, since they are at risk of incurring in psychopathological symptoms, which have a negative effect on their QoL. Secondly, these findings suggest that psychological factors should be treated during rehabilitation programs. Preliminary studies have evaluated the feasibility and the effects of psychological treatments specifically tailored for patients with acromegaly, but this field of study is still in its infancy [26, 27].

Concluding, we report a high prevalence of depression among patients with acromegaly, which is associated with female sex and especially arthropathy, with which independently impairs patients’ quality of life. Also, considering the finding of suicides among acromegalic subjects with a high AIMS depression score, it is always necessary to investigate the mood and anxiety of patients. Further studies could confirm the usefulness of depression scales in the screening of depression in acromegaly.

## Supplementary Information

Supplementary Information

## Data Availability

The data that support the findings of this study are available from the corresponding author upon reasonable request.
