# Supplementary material for: Psychological complications in patients with acromegaly: relationships with sex, arthropathy, and quality of life
Source: Endocrine. 2022 Jul 2;77(3):510–8. doi: 10.1007/s12020-022-03106-8 (PMC9385810; doi:10.1007/s12020-022-03106-8)
Supplement: Supplementary file 1 — Supplementary Information [file 12020_2022_3106_MOESM1_ESM.docx]

We thank the reviewer for her/his careful revision of our paper, highlighting several points of improvement for our manuscript, and allowing us to clarify reasonable doubts that could have arisen in the readers.

1 WOMAC is a rapid and easy to use tool to effectively evaluate the articular pain and physical functionality and performance. Did you evaluate the negative impact on this score of skeletal and osteoporotic complications such as history of hip and/or presence of vertebral fractures? I suggest that these complications could surely and negatively influence some of the WOMAC parameters. Moreover, in a very recent published study, vertebral fractures were reported as a highly prevalent complications also in patients with a recent diagnosis of acromegaly (DOI: 10.1210/clinem/dgac183), suggesting that also in your cohort, these complications could influence the patients WOMAC scores and, consequently, their depression scores and qol. It might not be a coincidence that were not observed differences regarding AIMS depression scores between patients with acromegaly and those with low back pain, probably caused, at least in part, also by vertebral fractures. Please, I suggest to comment this point. Thanks.

The point is well taken. The patients of our cohort were only in part studied with vertebral morphometry, and a homogeneous evaluation in this retrospective multicentric setting is not available. We now report this consideration, and the suggested reference, at page 12 lines 283-285, since vertebral fractures are for sure an element that could contribute to worse WOMAC scores leading to QoL impairment.

2 please, although Standardized IGF at observation resulted as a non-significantly factor associated with AIMS and qol scores in univariate analyses, did you evaluate the differences between patients with controlled and active diseases regarding the qol, AIMS and WOMAC scores? Did you evaluate to include in univariate and multivariate analyses for AIMS and qol the disease status (as active vs controlled) instead of IGF values? Thanks.

Another well taken point. When studying the determinants of worse depression AIMS scores, we performed the analyses also according to disease status as a categorical variable (controlled vs active disease), with no significant difference. We chose the continuous IGF1 data for the multivariate analysis reported in table 2 in order to keep only one of two strictly dependent variables (IGF1 sds and active disease). In the revised version we state that the use of disease activity instead of IGF1 SDS did not change the results (page 10, lines 211-214).

3a please, I suggest to report in your results in details the time interval from diagnosis to study enrollment, and, if it is possible, the time interval from diagnosis to controlled disease therapeutical achievement, specifying, in methods section, the therapeutical approaches criteria used in your patients.

We thank the reviewer for this suggestion, which helps us to improve the manuscript. We now report the mean time from diagnosis, and of active disease with SDS (page 10, lines 211-214). We now state as requested, in the methods section (page 6, lines 131-135), that the therapeutic approaches were chosen by the different physicians according to their expertise, with no influence from this study since it is observational. Yet surgery was the first line therapy in the 52.6 % of patients, SSA in the 36.2% while other therapies and RT were only adopted when the first line treatments weren't effective or viable. In 11.2% of patients, it is not known whether it was initially performed surgery or medical therapy.

3b Did you evaluate to include in univariate and multivariate analyses for AIMS and qol the time interval from diagnosis to controlled disease therapeutical achievement (using for patients with active disease the entire time interval from diagnosis to study enrollment) instead of the time interval from diagnosis to study enrollment? Thanks.
Once again, we thank the reviewer for this important statement. Actually, we initially studied both the time from diagnosis and the time of active disease (none of them proved to be significant). Since we also evaluated the IGF1 sds, we decided to keep time lapsed from diagnosis in the multivariate analysis (did not change the results significantly). We now state that when using time of active disease instead of time lapsed from diagnosis, the significant results remained the same (page 10, lines 211-214).

4 please, i suggest to specify the cause of mortality. Thanks.

We thank the reviewer for this suggestion, which helps us to improve the manuscript. We now report the main causes of mortality in  supplementary table 2, as stated in page 9 line 201
